# Microbial synthesis of a useful optically active (+)-isomer of lactone with bicyclo[4.3.0]nonane structure

**DOI:** 10.1038/s41598-017-18876-9

**Published:** 2018-01-11

**Authors:** Filip Boratyński, Agata Janik-Polanowicz, Ewa Szczepańska, Teresa Olejniczak

**Affiliations:** Department of Chemistry, Wroclaw University of Environmental and Life Sciences, Wrocław, 50375 Poland

## Abstract

Lactone **2a** of a bicyclo[4.3.0]nonane structure is a good starting material for synthesis of many attractive compounds. Enantiomerically enriched (−)-(3a*R*,7a*S*)-lactone **2a** is produced by whole cells of bacteria. In order to examine the impact of the absolute configuration on biological activity we evaluated the process affording the opposite isomer. To this purpose *Candida pelliculosa* ZP22 characterized by high dehydrogenase activity was used. The goal of presented work was to perform bioreactor scale microbial one-pot oxidation of diol with selected yeast strain *C*. *pelliculosa* ZP22 to obtain chiral (+)-(3a*S*,7a*R*)-lactone **2a**. The idea was to influence on alcohol dehydrogenase activity by increasing the activity of pro-(+)-ADH and simultanously diminishing the activity of pro-(−)-ADH. The optimization of biotransformation conditions involved the manipulation of the nutritional and physical parameters. Selection of the optimal medium in order to improve yield and process enantioselectivity was based on a two-level factorial design methodology. We have also studied the relationship between microbial growth and biosynthesis of lactone **2a**. Preparative oxidation of diol **3a** (400 mg/L, 2.9 mM) catalyzed by *C*. *pelliculosa* ZP22 in an optimized conditions afforded enantiomerically enriched (+)-(3a*S*,7a*R*)-isomer of lactone **2a** with the isolated yield (30%).

## Introduction

Asymmetric transformations catalyzed by whole cells of microorganisms or isolated enzymes have become an attractive alternative for traditional methods leading to optically pure compounds, which derive from either natural sources or by organic synthesis^1–4^. It is particularly important in the synthesis of biologically active compounds in which biological activity usually depends on the absolute configuration in a molecule^5,6^. Therefore, a growing need to find new biocatalysts for synthesis of optically pure molecules with various biological activities is of high importance in the current chemistry. To achieve this purpose in the field of the synthesis of lactones two biocatalytic strategies: kinetic resolutions^7–9^ and stereoselective reactions^10–14^ were applied so far.

Previously Boratyński^12,14–16^ presented a one-pot biotransformation of diols to chiral lactones. This method involves an asymmetric synthetic value leading to lactones that are well-known to be attractive chiral building blocks. Currently we are particularly interested in the development of a stereoselective biooxidation, which will be significant in the multi-step synthesis of optically active lactones of a bicyclo[4.3.0]nonane structure.

Compounds of such structure represent a large group of natural phtalide derivatives^17^. They have been isolated from plants of the *Apiaceae* family Lindl. (*Ligusticum officinale* (Loveroot, old English Lovage), *L*. *chuanxiong*, *L*. *wallichii* (Chinese Lovage), *Angelica sinensis* (Chinese Angelica), *Apium graveolens* (celeriac) and *Petroselinum crispum* (parsley)) used in herbal medicine, especially in Chinese folk medicine. More than 70 structures of these lactones were documented. The advantage of this group of compounds is a broad spectrum of biological activity, such as insecticidal^18,19^, fungicidal^18^, fragrance^20^, antioxidant^21^ and anticoagulant^22^, anti-proliferative^23^, cytotoxic^24^. However, obtaining these valuable natural compounds directly from a plant material is inefficient and thus uneconomic. Chemical synthesis, although efficient, does not recommend by the green chemistry. Alternative approach providing to the optically pure isomers is biocatalysis.

It is worth mentioning that whole cells of yeast are well-known biocatalysts. They catalyze reduction reactions of a carbonyl group^25–28^ and a carbon-carbon double bond^29,30^, hydrolase reaction^31^ and formation of a carbon-carbon double bond^32,33^. Additionally, reports published on oxidation reactions performed by yeast are not commonly encountered. Yeast alcohol oxidases were proved to be responsible for the oxidation reaction of some primary^34^ and secondary alcohols^35^, sulfides^36^, racemization^37^ and deracemization reactions^38^. It is a well-known fact that whole-cell yeast are highly applicable due to the numerous advantages of their application. Yeast cells are mainly nonpathogenic, inexpensive and can be stored in dried form for a very long time. Yeast, compared to other biocatalysts, are simple to grow (higher increase in biomass) on cheap carbon sources (lower nutritional requirements). One of the major advantages of biotransformation via whole-cells is the availability of all necessary cofactors so it makes ineffective to apply a cofactor-regeneration system. Furthermore, whole-cells yeasts are well-protected within their natural cellular environment, which makes the catalytic system more stable. However, employing wild-type yeast strains as whole-cell biocatalysts also imputes some limitations one of which is the presence of a large number of different dehydrogenases, which quite often overlap in substrate specificity.

**Figure 1:**
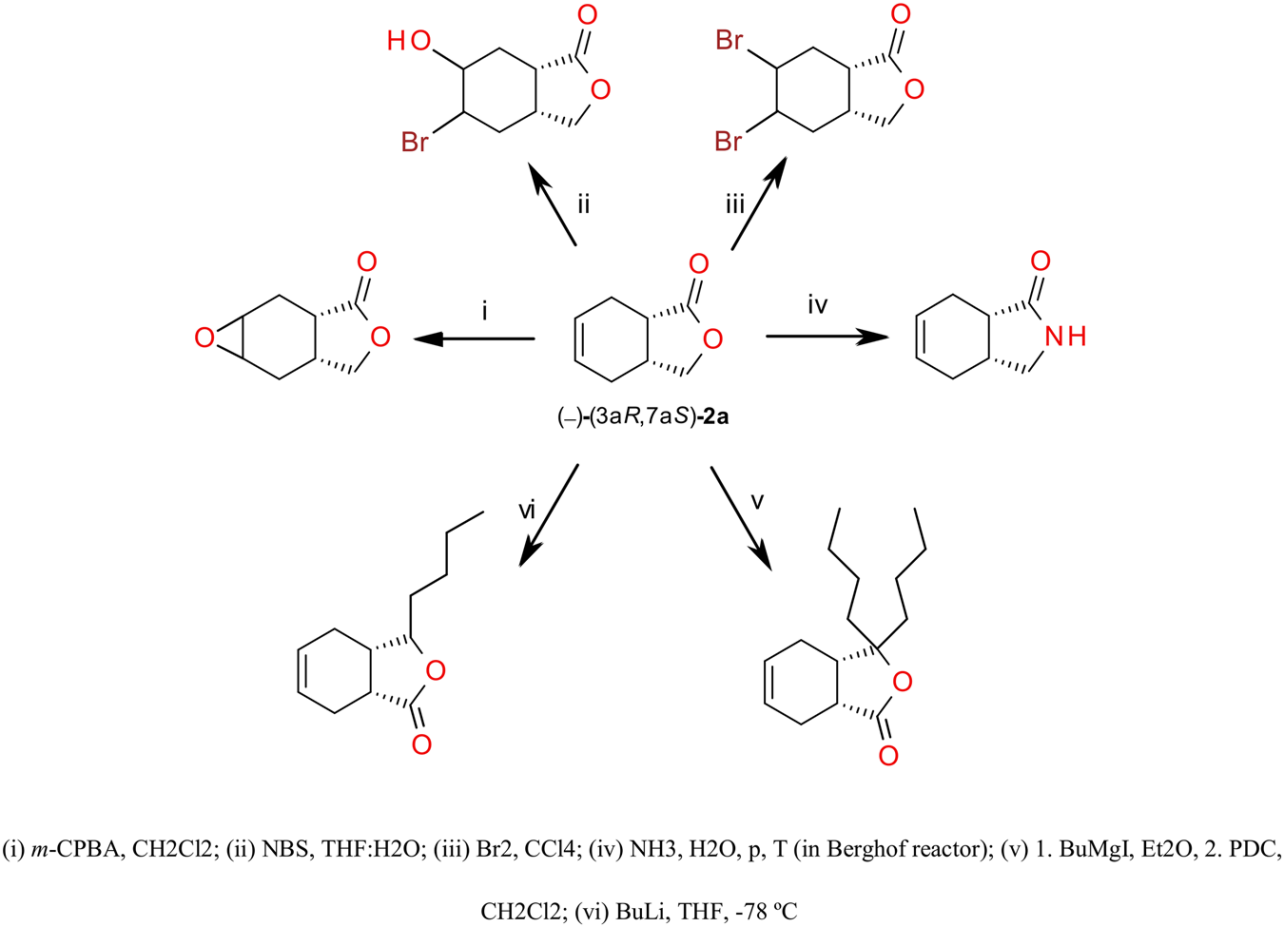
Lactone **2a** as a useful synthon in the synthesis of a broad range of biologically active compounds.

Lactone **2a** of a bicyclo[4.3.0]nonane structure is a good starting material for synthesis of many attractive compounds. Olejniczak^39,40^ synthesized a wide range of biologically active racemic derivatives, among them phtalide lactones, epoxy lactones and derivatives as well as lactams and their derivatives (Fig. 1). Many of them indicate high fungistatic activity against *Aspergillus glaucus*, *Botrytis cinerea* and *Penicillum citrinum*.

With application of enzymes Walczak^39^ obtained (−)-(3a*R*,7a*S*)-lactone **2a** and its chiral derivatives, which exhibit higher fungistatic activity than its racemates. Boratyński^15^ results showed that microbial oxidation of *meso* diol **3a** involving bacterial whole cells afforded enantiomerically enriched (−)-(3a*R*,7a*S*)-lactone **2a** as well. *Micrococcus sp*. DSM 30771 was the most effective (lactone **2a** isolated yield 38%, ee = 94%) among tested biocatalysts. However, in order to examine the impact of the absolute configuration on biological activity we need to evaluate the process affording the opposite isomer. Among tested yeast *Candida pelliculosa* ZP22 was selected to produce (+)-(3a*S*,7a*R*)-isomer of lactone **2a** with modest enantioselectivity^41^. The goal of this study is to carry out the bioreactor scale yeast transformations focusing on process enantioselectivity and (+)-(3a*S*,7a*R*)-lactone **2a** yield improving. Development of stereoselective biooxidation step will be crucial in synthesis of chiral lactones as chiral building blocks in various asymmetric syntheses.

Many methods were employed in order to improve the selectivity of whole cell yeast biotransformation. These methods involved modifications in cultivation conditions with the application of different carbon and nitrogen sources and the use of two-phase systems^42^ or application of ionic liquids^43^. It stands to reason that the optimization processes are crucial for industrial production scale. Thus we have taken up research on improvement in productivity of the microbial secondary metabolite via manipulation of both nutritional and physical parameters. In our present study, based on a fractional factorial experimental design^44,45^, the cultivation of *C*. *pelliculosa* ZP22 was optimized. We have been also studied the relationship between microbial growth and biosynthesis of lactone.

## Materials and Methods

### Analysis

^1^H NMR and ^13^C NMR spectra were recorded in CDCl_3_ solutions on a Bruker Avance^TM^ 600 (600 MHz, Billerica, MA, USA) spectrometer. IR spectra were determined on a FT-IR Thermo-Nicolet IR300 (Waltham, Ma, USA) infrared spectrometer. Molecular mass was confirmed on a Varian Chrompack GC MS CP-3800 Saturn 2000 GC/MS/MS with ionization energy of 70 eV, using HP-1 column (crosslinked methyl silicone gum, 25 m × 0.32 mm × 0.25 μm film thickness) and HRMS analysis were conducted on a micrOTOF-Q Bruker. Gas chromatography analysis (GC, FID, carrier gas H_2_) was carried out on an Agilent Technologies 7890 N (GC System, Santa Clara, CA, USA) with application of a chiral column CP7502 Chirasil-Dex CB (25 m × 0.25 mm × 0.25 μm) with the following temperature program: 80 °C, 150 °C (4 °C/min), 200 °C (20 °C/min) (1 min). The total run time was 21 min. The following retention times of each enantiomers of lactone **2a** were established: *t*_R_ (+)-(3a*S*,7a*R*)-**2a** = 15.77 min and *t*_R_ (−)-(3a*R*,7a*S*)-**2a** = 15.86 min. Optical rotation was measured on an Autopol IV automatic polarimeter (Rudolph, Hackettstown, NJ, USA) in chloroform solutions (concentration: g/100 mL). Analytical TLC technique (SiO_2_, DC-Alufolien Kieselgel 60 F254, Merck) were performed by using methylene chloride:methanol (95:5). A solution of 1% Ce(SO_4_)_2_ and 2% phosphoromolybdenic acid in 10% H_2_SO_4_ was used as a visualizing agent. Preparative column chromatography (SiO_2_, Kieselgel 60, 230–400 mesh, 40–63 μm, Merck) was performed by using methylene chloride:methanol (95:5).

### Chemicals

*cis*-4-Cyclohexene-1,2-dicarboxylic anhydride (**1a**), CelLytic^TM^ Y and LiAlH_4_ were purchased from Sigma-Aldrich Chemical Co. (St. Louis, MO, USA).

### Reduction of anhydride 1a

A solution of *cis*-4-cyclohexene-1,2-dicarboxylic anhydride (**1a**) (6 mmol) in a mixture of diethyl ether (20 mL) and tetrahydrofuran (10 mL) was added dropwise to LiAlH_4_ (8 mmol) in diethyl ether (20 mL). The mixture was stirred for 16 hours under reflux. When the reaction was completed (controlled by GC, TLC), water was added to decompose the excess of LiAlH_4_. The mixture was then acidified with 0.1 M HCl and the products were extracted with chloroform. Then the extract was washed with saturated NaCl and dried over anhydrous MgSO_4_. The crude products were purified by column chromatography (silica gel, methylene chloride:methanol, 95:5) resulting 73.1% of *cis*-4,5-bis(hydroxymethyl) cyclohexene (**3a**) and 7.5% of *cis*-3a,4,7,7a-tetrahydro-1(3 *H*)-isobenzofuranone (**±**)-(**2a**). The spectral data of obtained products were presented below.

*cis*-3a,4,7,7a-Tetrahydro-1(3 *H*)-isobenzofuranone (**±**)-(**2a**): ^1^H NMR (500 MHz, CDCl_3_) δ: 1.80–1.96 (m, 1 H, one of CH_2_–4), 2.19–2.54 (m, 3 H, one of CH_2_-4, H-3a, one of CH_2_-7), 2.55–2.81 (m, 2 H, one of CH_2_-7, H-7a), 4.00 (dd, 1 H, *J* = 8.8, 2.0 Hz, one of CH_2_-3), 4.30 (dd, 1 H, *J* = 8.8, 5.1 Hz, 1 H, one of CH_2_-3), 5.66–5.78 (m, 2 H, H-6, H-5); ^13^C NMR (151 MHz, CDCl_3_) δ: 21.9 (CH_2_-4), 24.6 (CH_2_-7), 31.9 (CH-7a), 37.2 (CH-3a), 72.7 (CH_2_-3), 124.8 (CH-5), 125.1 (CH-6), 179.1 (C=O); IR (film, cm^−1^): 1771 (s); GC-EIMS: 138 (M + 1).

*cis*-4,5-Bis(hydroxymethyl)cyclohexene (**3a**): ^1^H NMR (500 MHz, CDCl_3_) δ: 1.96-2.08 (m, 4 H, CH_2_-6, CH_2_-3), 2.09-2.17 (m, 2 H, CH-1, CH-2), 3.18 (s, 2 H, 2xOH), 3.57 (m, 2 H, CH_2_-OH), 3.70 (m, 2 H, CH_2_-OH), 5.59 (s, 2 H, CH-5, CH-4); ^13^C NMR (151 MHz, CDCl_3_) δ: 26.92 (CH_2_-3, CH_2_-6), 37.79 (CH_2_-1, CH_2_-2), 64.03 (CH_2_-OH), 125.52 (CH-5, CH-4).

### Microorganism

*Candida pelliculosa* ZP22 came from Department of Biotechnology and Food Microbiology at Wroclaw University of Environmental and Life Sciences (Poland). It was maintained at 4 °C on Sabouraud agar slants containing peptone (10 g), glucose (40 g) and agar (15 g) dissolved in water (1 L) at pH 5.5.

### Growth conditions

The composition of culture media (g/L H_2_O): **P**: 30 g glucose, 10 g peptone; **A**: 40 g glucose, 15 g (NH_4_)H_2_PO_4_, 7 g KH_2_PO_4_, 0.8 g MgSO_4_ × 7H_2_O, 0.1 g NaCl, 0.06 g ZnSO_4_ × 7H_2_O, 5 × 10^−3^ g CuSO_4_ × 5H_2_O, 0.01 g MnSO_4_ × 4H_2_O; **B**: 50 g glucose, 7 g (NH_4_)H_2_PO_4_, 3.5 g KH_2_PO_4_, 0.12 g ZnSO_4_ × 7H_2_O, 1 g MgSO_4_ × 7H_2_O, 0.025 g NaCl, 0.02 g MnSO_4_ × 4H_2_O, 0.01 g CuSO_4_ × 5H_2_O.

Conditions of experiments performed in preparative scale: **P25**: 25 °C, **P** medium; **A25**: 25 °C, **A** medium; **A30**: 30 °C, **A** medium; **A35**: 35 °C, **A** medium; **B35**: 35 °C, **B** medium.

### Media optimization

The values of the gradient shown in Table 1 were used to conduct experimental optimization. At this stage, eight experiments were performed (Table 2). The starting point in this stage of research was the middle point of the previous plan, where variables were 0 (control). In the second stage of optimization, experiments in the directions designated by the gradients of each function were performed (Table 3). The way in which values of the variables were accepted, was analogous to initial experiment, where the 0 value comprised the medium composition, in which the highest conversion was observed (Table 4). The concentration plan of each component assumed either an increase or a decrease in the output value of ±50% (or +100% in cases of small amounts of several salts). The hydrated sulfate salts were treated together as one component.

**Table 1:** Real values for each variables in the 1^st^ stage of factorial design.

Components	Level		
	L^a^	0	H^b^
	Content of components [g/L]		
(NH_4_)H_2_PO_4_	7.5	15	22.5
KH_2_PO_4_	3.5	7	10.5
Glucose	20	40	60
NaCl	0.05	0.1	0.2
**Minerals**			
CuSO_4_ × 5H_2_O	2.5 * 10^−3^	5 * 10^−3^	0.01
ZnSO_4_ × 7H_2_O	0.03	0.06	0.12
MnSO_4_ × 4H_2_O	5 * 10^−3^	0.01	0.02
MgSO_4_ × 7H_2_O	0.4	0.8	1.2

^a^Low-content; ^b^High-content.

**Table 2:** Conversion of **3a** in each variant with coded values for five variables in the 1^st^ stage of factorial design.

Components						
Variant	(NH_4_)H_2_PO_4_	KH_2_PO_4_	Glucose	NaCl	Minerals	Conversion
1	H	H	H	H	H	++
2	L	H	H	L	H	+++
3	H	H	L	L	L	+
4	L	H	L	H	L	+
5	H	L	H	H	L	+
6	L	L	H	L	L	+++
7	H	L	L	L	H	+++
8	L	L	L	H	H	+

^a^Low-content; ^b^High-content; + weak; ++ good; +++ very good.

**Table 3:** Real values for each variables in the 2^nd^ stage of factorial design.

Components	Level		
	L^a^	0	H^b^
	Content of components [g/L]		
(NH_4_)H_2_PO_4_	5	7.5	12
KH_2_PO_4_	3.5	7	10.5
Glucose	50	60	70
NaCl	0.025	0.05	0.05
**Minerals**			
CuSO_4_ × 5H_2_O	0.01	0.01	0.02
ZnSO_4_ × 7H_2_O	0.12	0.12	0.24
MnSO_4_ × 4H_2_O	0.02	0.02	0.04
MgSO_4_ × 7H_2_O	0.8	1	1.2

^a^Low-content; ^b^High-content.

**Table 4:** Conversion of **3a** in each variant with coded values for six variables in the 2^nd^ stage of factorial design.

Variant	Components						
	(NH_4_)_2_HPO_4_	KH_2_PO_4_	Glucose	NaCl	Minerals	MgSO_4_	Conversion
1	L	L	L	H	L	H	+++
2	H	L	L	L	H	H	++
3	L	L	H	L	L	L	++
4	H	L	H	H	H	L	+
5	L	H	L	H	L	L	+
6	H	H	L	L	H	L	+
7	L	H	H	L	L	H	+
8	H	H	H	H	H	H	+

^a^Low-content; ^b^High-content; + weak; ++ good; +++ very good.

The experiments for media optimization were performed in shaken flasks containing sterile culture medium (50 mL) with the composition prepared according to Tables 2 and 4. After inoculation by *C*. *pelliculosa* ZP22, they were incubated in an orbital shaker (140 rpm, 25 °C) until late exponential phase, followed by induction of **3a** (0.04 g/mL). The biotransformation progress was followed by gas chromatography applied with chiral column. The control experiments, without microorganism, of diol **3a** and lactone **2a**, indicating their stability in aqua solution, were performed.

### The cultivation of *C*. *pelliculosa* ZP22

The reactor used to run the fermentations was a 3 L New Brunswick Scientific BioFlo III (Brunswick, Ramsey, MN, USA). The temperature and agitation were maintained at 25 °C or 35 °C and 600 rpm, respectively. A Broadley James D100 Series Oxyprobe was used to track the dissolved oxygen level to ensure the system was not oxygen transfer limited. The air flow rate into the reactor was 1 L/min and was passed through a sterile 0.2 μm hydrophobic fluoropore PTFE filter. The reactor containing medium was sterilized at 121 °C for 25 minutes prior to use.

A 250 mL Erlenmeyer flask containing sterile culture medium (100 mL), was inoculated by *C*. *pelliculosa* ZP22 and incubated in an orbital shaker (140 rpm, 25 °C) until late exponential phase. The content of pre-culture flask was aseptically poured into a final volume of 1500 mL and the culture was grown until the biomass concentration had reached OD_600_ 0.4-0.6. With respect to the screening scale experiments performed previously, 0.6 g of diol **3a** diluted in 5 mL of acetone was used in the preparative method. The substrate **3a**, was supplied through a port on the reactor lid using sterile pipet tips to reduce the possibility of contamination. The air flow was continuously supplied throughout the duration of the transformation. The cell growth was monitored every 2 hours (in lag and mid exponential growth phase) and every 24 hours (in late exponential and stationary growth phase) by measuring OD_600_. The 11 day biotransformation progress was followed by gas chromatography applied with chiral column. The reaction mixture was divided in three portions (3 × 500 mL), acidified by 0.1 M HCl, washed with brine and extracted overnight with ethyl acetate (3 × 500 mL) on laboratory shaker. After extraction, centrifuged (10,000 rpm, 20 mins) and evaporated. The crude product was purified by column chromatography using a mixture of hexane/acetone (3:1) as a mobile phase. Oxidation of diol **3a** (0.6 g) after 11 days in experiment **B35** gave 0.177 g (30% yield) of (+)**-**(3a*S*,7a*R*)**-2a**, ee = 70% (${[{\rm{\alpha }}]}_{589}^{25}=+55.4^\circ$ (c = 1.0, CHCl_3_), ref.^23^ ${[{\rm{\alpha }}]}_{589}^{25}=-67.1^\circ$ (c = 1.0, CHCl_3_), ee = 100%). The spectroscopic and chromatographic data, which confirm lactone **2a** structure were attached as supporting information (Supplementary Figures S1–S5).

### Biomass determination

Determination of the living yeast cells on the basis of serial dilution count method was conducted. Briefly, 1 mL of each sample was serially diluted tenfold in 9 mL of sterile physiological saline. Dilutions from 10^−9^ to 10^−16^ were spread in triplicates on Sabouraud agar medium. All inoculated plates were incubated at 25 °C for 48 hours. After incubation, colonies appeared on each plate were counted taking into considerations their morphological characteristics.

Total cellular growth was determined by measuring optical density (OD_600_). The optical density was previously correlated with the quantity of the dry cells, data not shown. To monitor the number of cells and strain, physiological data automated instrument Scepter™ cell counter was applied as well.

### The crude extract preparation

Samples (10 mL) from the bioreactor were withdrawn every 2 or 24 hours. Cells from culture were harvested by centrifugation for 10 min at 15.000 rpm and washed with 100 mM Tris-HCl buffer (pH 8.5). The pellets were suspended in 10 mL of the same buffer containing 1 mM DDT and 100 μM PMSF, mixed and centrifuged for 10 min at 15.000 rpm. In order to extract the proteins cell, lysis agent CelLytic^TM^ Y was applied. Cell debris was removed by centrifugation for 10 min at 15.000 rpm. The supernatant was used for further studies.

### Protein determination

Protein concentration was determined according to Bradford method^46^ using bovine serum albumin as calibration standard.

### Activity assay based on reduction of NAD^+^

Dehydrogenase activity was determined by following the increase in absorbance at 340 nm using 1 mM of **3a** as substrate dissolved in 100 mM Tris-HCl buffer (pH 8.5). Enzyme activity was calculating by measuring the formation of NADH-H^+^ at 340 nm. One unit of the enzyme activity was defined as the amount of enzyme required to reduce 1 μmol of NAD^+^ per minute.

### Activity assay based on reduction of TTC

Dehydrogenase activity was determined by the reduction of a colorless 2,3,5-triphenyltetrazolium chloride (TTC) to a colorful 1,3,5-triphenyltetrazolium formazan (TPF) at 485 nm by following a modified TTC assay^47^. Activity was expressed in units, where one unit corresponds to the release of 1 μmol of TPF protein per minute.

## Results and Discussion

**Figure 2:**
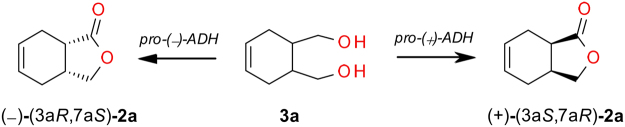
One-pot microbial oxidation of *meso* diol **3a** to both isomers of lactone **2a**.

Biological properties of chiral compounds are many a time related to absolute configuration. Therefore, we are especially interested in elaboration of biotechnological methods of synthesis both enantiomers of lactone (Fig. 2). In this microbial oxidation both alcohol dehydrogenases (*pro-*(+)*-ADH* and *pro-*(−)*-ADH*) take part. Our idea was to increase the activity of *pro-*(+)*-ADH* and simultanously diminish activity of *pro-*(−)*-ADH* by manipulation of *C*. *pelliculosa* ZP22 biotransformations conditions. Modifications of performed experiments concerned mainly cultivation conditions, particularly type of medium and process temperature. The effect of the culture medium composition optimized in a two-level factorial design on the conversion rate and enantiomeric excess of lactone **2a** was studied. We analyzed the effect of temperature on abovementioned parameters as well. Moreover the amount of living cells and enzyme activity in order to accurately investigate the whole process was also monitored.

**Figure 3:**
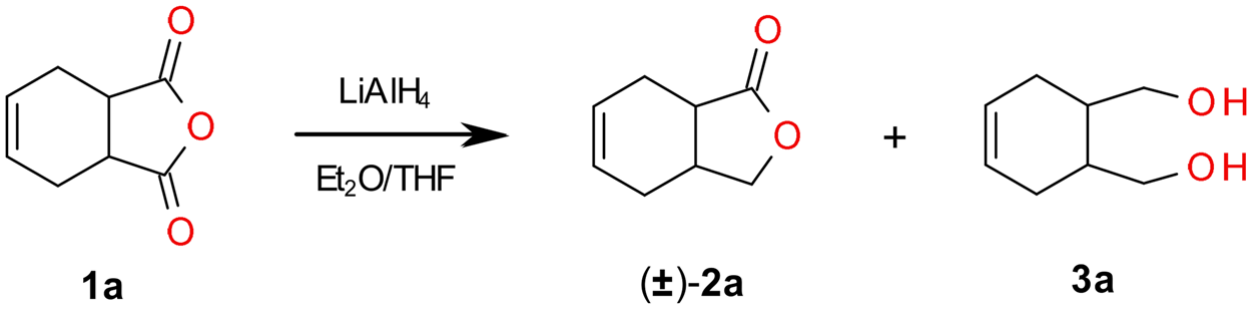
Chemical reduction of anhydride **1a**.

The substrate for biotransformation *meso* diol **3a** was obtained in a good yield in the reduction process with lithium aluminum hydride of the corresponding low-cost anhydride **1a** (Fig. 3). Additionally, a small amount of lactone **2a** in the racemic form was isolated and used in a gas chromatography analysis to establish retention time of both enantiomers of lactone **2a**.

### The effect of biotransformation conditions on content and enantiomeric excess of lactone 2a

Transformations of diol **3a** by *C*. *pelliculosa* ZP22 were conducted for 11 days since enantiomeric excess of lactone **2a** was higher than ee = 60%. Bioreactor scale experiments were performed with the following standardized parameters: medium volume (1.5 L), aeration rate (1 v/m), stirring speed (600 rpm) and pH. Samples from bioreactor were withdrawn every 24 hours in order to monitor the progress and enantioselectivity of the biotransformation by chiral gas chromatography (CGC) and to determine both the level of biomass and enzyme activity.

Initially the bioxidation process was performed in two types of media. It is sometimes observed that minimal medium induces microorganisms to produce enzymes able to transform xenobiotics. Firstly, to test scaling-up methodology, the biotransformation was performed in Sabouraud medium in 25 °C (**P25**) in the same conditions as in the screening scale. The second experiment (**A25**) was set up in enriched medium (**A**) chosen on the basis of the literature data describing the growth of yeast of the *Candida* genera^48^.

**Figure 4:**
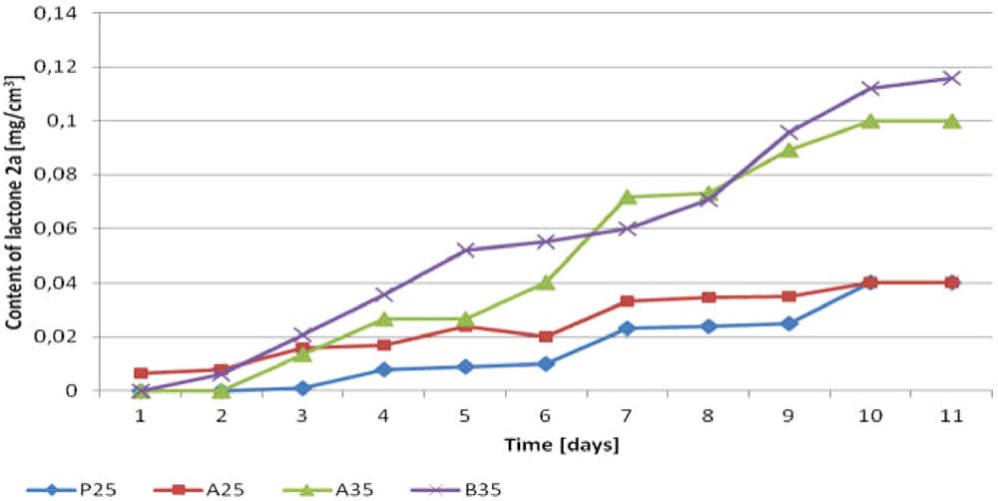
Effect of different biotransformation conditions (**P25**, **A25**, **A35**, **B35**) on the content of lactone **2a** (according to GC) in the course of bioreactor scale experiments.

**Figure 5:**
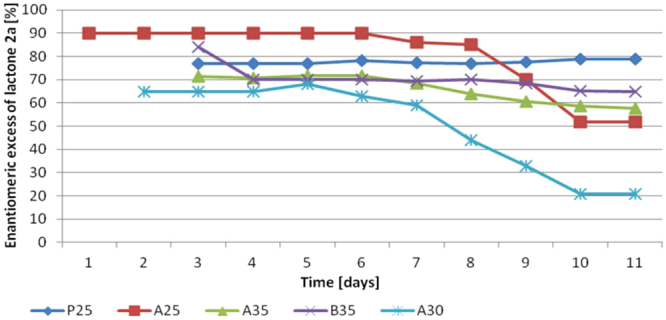
Effect of different biotransformation conditions (**P25**, **A25**, **A30**, **A35**, **B35**) on the enantiomeric excess of lactone **2a** (according to chiral GC) in the course of bioreactor scale experiments.

As it can be observed in Fig. 4 lactone **2a** was formed from the 3^rd^ day of biotransformation in the **P25** as opposed to **A25**, where the product was formed just after substrate addition. The conversion of diol **3a** in both experiments was low. Application of different media did not affect on diol **3a** conversion significantly. Nevertheless, in the **P25** the modest enantiomeric excess (ee = 78.8%) of lactone **2a** was observed (Fig. 5). It is worth pointing out that the enantioselectivity of the whole process was at constant level. In contrast, a decrease of enantioselectivity from ee = 90% to ee = 52% in **A25** was observed. Our goal was to obtain lactone **2a** with the highest purity, so we decided to activate *pro-*(+)*-ADH* in enriched medium (**A**) since we initially observed high enantioselectivity (ee = 90%) of biotransformation.

**Figure 6:**
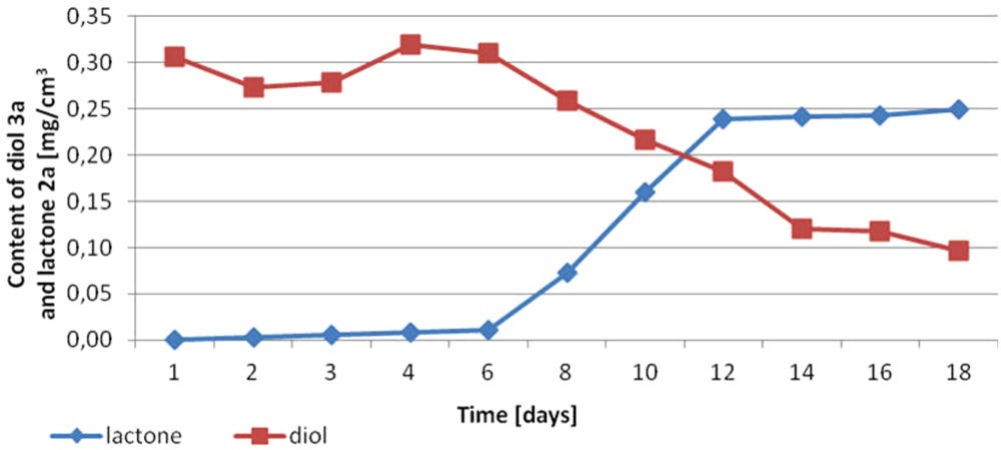
The content of diol **3a** and lactone **2a** (according to GC) in the course of bioreactor scale experiment (**A30**).

Significantly low amount of lactone **2a** in both experiments (**P25** and **A25**) encouraged us to investigate whole process. Therefore preparative biotransformation **A30** was conducted for 18 days unless no more lactone **2a** was formed (Fig. 6). Increasing the time of oxidation caused higher conversion of diol **3a** however with lower optical purity of lactone **2a** (ee = 33%) in comparison to other experiments (**P25**, **A25**, **A35**, **B35**). Such low enantiomeric excess of lactone **2a** has proven that both, *pro-*(+)*-ADH* and *pro-*(−)*-ADH* were active during biotransformation.

From our experience appears that generally higher temperature of microbial transformations causes decrease in enantioselectivity, however it can stimulate the biomass growth. In the next experiment (**A35**) the temperature of biotransformation was increased from 25 °C to 35 °C. As expected, higher temperature applied in **A35** caused more than a twofold increase in the amount of lactone **2a** (Fig. 4). Unfortunately, despite the initially sustained level of enantiomeric excess (ee = 70%) between 3-7 day, the further conversion of diol **3a** caused increase of *pro-*(−)*-ADH* activity, which afford in decrease of process enantioselectivity (ee = 60%) (Fig. 5).

The last modification (**B35**) concerned the application of the improved culture medium (**B**). The choice of the optimal culture medium was performed in the screening scale experiments on the basis of a two-level factorial design. In the experiment with optimized medium enantiomerically enriched lactone **2a** (ee = 70%) (Fig. 5) with 30% of isolated yield (Fig. 4) was achieved. The spectroscopic and chromatographic data of obtained lactone **2a** were attached as supporting information (Supplementary Figures S1–S5).

### The effect of biotransformation conditions on the number of cells

To determine the influence of the type of medium and the process temperature on *C*. *pelliculosa* ZP22 growth a series of experiments, allowing measurements of the number of cells, were provided. The total cell number was measured on the basis of optical density measurements (OD_600_) and the use of Scepter^TM^ cell counter, which provided additional data about cell size and potential contamination of the yeast culture. Measurements of living cells were based on a serial dilution method, which assumes inoculation of petri dishes and subsequent counting of grown colonies. The samples were taken just after substrate addition from the late logarithmic growth phase.

**Figure 7:**
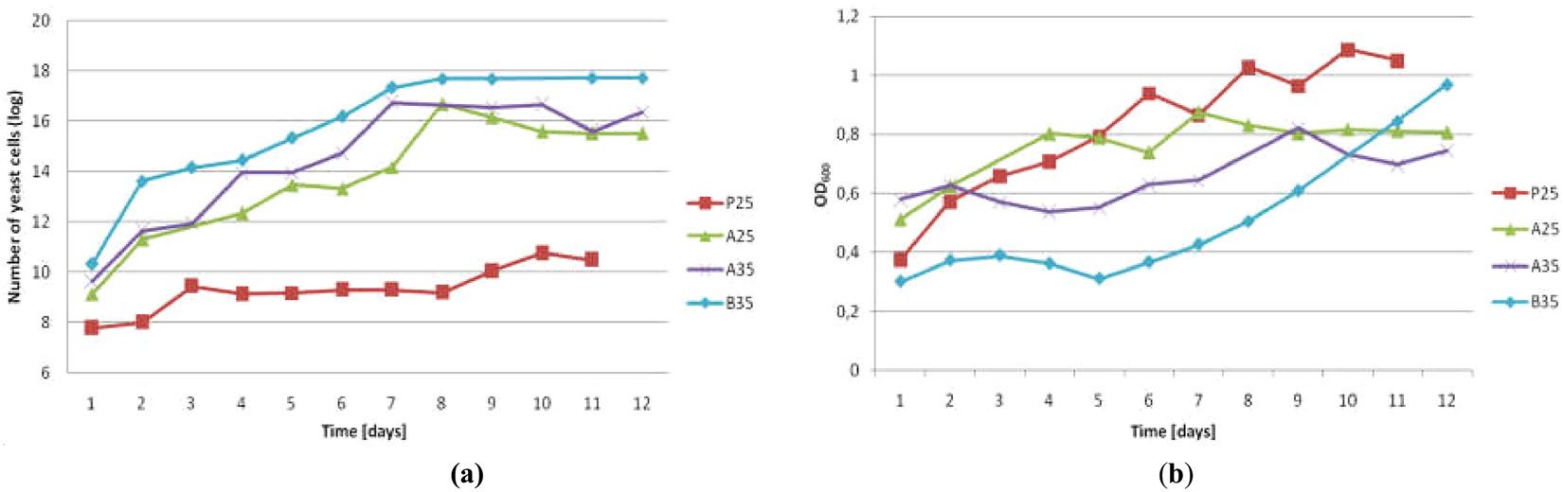
The number of living cells (**a**) and optical density profile (**b**) in the course of bioreactor scale experiments (**P25**, **A25**, **A35**, **B35**).

The total number of cells was the highest in **P25** (Fig. 7b). However, the number of living cells was at significantly lower level (Fig. 7a) by having compared it to all experiments carried out in enriched media. Until the 8^th^ day the cells amount was 10^9^ and then increased to 10^11^. In contrary, cultivation on enriched media (**A25**, **A35**, **B35**) caused a steady increase of the number of living cells from 10^9^ to 10^17^.

A slight increase in cells number in **P25** and an inconsiderable increase of the amount of lactone **2a** were observed. On the other hand, an increase of the number of cells in the **A** media, particularly in **A35** and **B35**, improved biotransformations significantly. Initially, when the number of cells was below 10^13^ (1–3 days), **2a** was not observed. Only when a further increase in biomass occurred, diol **3a** was converted into lactone **2a**. Stabilization of cells number caused the lack of further conversion of **3a**. In **A25** the relationship between cells number (Fig. 7) and an increase of **2a** (Fig. 4) showed that the amount of lactone **2a** doubled between 6–10 day, which was correlated with an increase of biomass from 10^13^ to 10^16^.

The conversion of diol **3a** depended on microbial growth, which was directly related to the composition of the cultivation medium. The life-time of *Candida* cells in **P** medium was shorter than in enriched **A** medium. Therefore, the more living cells produced in **A** medium, the higher conversion of diol **3a** was observed. The temperature of the biotransformation had less influence on the amount of living cells. Nevertheless, the cells in **B35** grew more rapidly, which afforded the highest quantity of the biomass.

### The effect of biotransformation conditions on the enzyme activity

The next part of the study involved measurements of the enzyme activity in different culture conditions and its correlation with the progress of the biotransformation. In this study two spectrophotometrical methods for dehydrogenases activity determination were used. The first one was based on measurements of the changes in absorbance during the reduction of the coenzyme NAD^+^ to NADH as a result of oxidation of diol **3a** to lactone **2a**. This selective method only defines the activity of dehydrogenases capable to catalyze the particular transformation. The second method involved the incubation of yeast biomass with a colorless substrate 2,3,5-triphenyltetrazolium chloride (TTC), which is enzymatically reduced to a colorful 1,3,5-triphenyltetrazolium formazan (TPF). This method was used to measure the total amount of all dehydrogenases.

**Figure 8:**
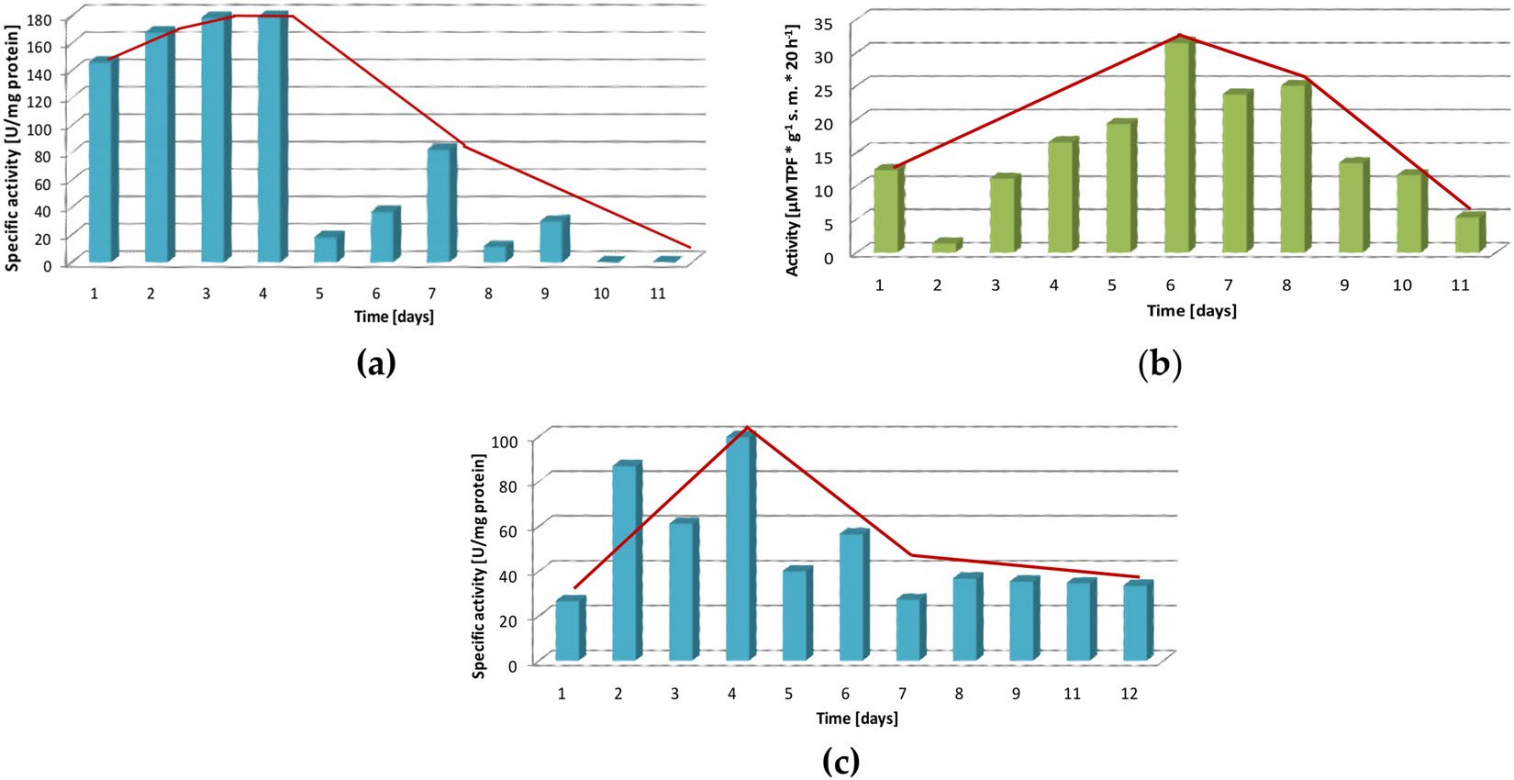
Dehydrogenase specific activity with tendency (red curve) based on NAD^+^ reduction in the course of bioreactor scale experiments (**a**) **P25**, (**c**) **B35**. Dehydrogenase activity with tendency (red curve) based on TTC reduction in the course of bioreactor scale experiment (**b**) **P25**.

Initially, the measurements of the enzyme activity were performed with both methods (Fig. 8a,b). In the method with a TTC the highest activity was observed between 4–9 day of the biotransformation, while in the method with a NAD^+^ the highest activity was between 1–4 day and then a decrease was subsequently observed at the end of the process. Differences in enzymatic activity can be explained by methods selectivity. In the first method all dehydrogenases produced by microorganism were measured, while in the second method only dehydrogenases responsible for oxidation of diol **3a**. In further experiments only a selective method with NAD^+^ was applied.

No correlation between initially high dehydrogenase activity (Fig. 8a) and low content of lactone **2a** established by GC (Fig. 4) was due to the formation of hydroxyaldehyde as an intermediate product in oxidation of diol **3a** to lactone **2a**. The low content of lactone **2a** in **P25** can be explained by decrease of activity of dehydrogenases responsible for the second step of oxidation. As Boratyński^12,14^ evaluated previously the pathway of microbial one-pot synthesis of lactones consists of two oxidation steps. In the first one, hydroxyl group of diol is oxidized and hydroxyaldehyde is formed. Further oxidation of hydroxyaldehyde, proceeding via hemiacetals, leads directly to lactone.

The increase of temperature and the use of optimized medium **B35** had a positive effect on the enzymatic activity (Fig. 8c). It should be pointed out that long-term and balanced distribution of activity in **B35** had its significant influence on the higher conversion of **3a**. The observed increase in cells number during the biotransformation, partially influenced the activity that increased to day 4 and then gradually decreased, but did not disappear in the last stage as it did in **P25**. It is also worth mentioning that in **P25** with the fewer number of cells, the activity was higher compared to **B35** in which cells number was higher with lower activity. A similar relationship between the activity of dehydrogenases and the number of cells was observed in the culture media optimization experiments performed on the basis of a two-level factorial design.

### Optimisation of culture media on the basis of a two-level factorial design

A two-level factorial experiment was applied to evaluate the effects of independent variables, namely the concentrations of components of medium **A**: glucose, (NH_4_)H_2_PO_4_, KH_2_PO_4_, MgSO_4_ × 7H_2_O, NaCl, and minerals (ZnSO_4_ × 7H_2_O, CuSO_4_ × 5H_2_O, MnSO_4_ × 4H_2_O) on lactone **2a** bioproduction (Table 1). In the first part of the experiment the highest conversion of **3a** was obtained in modifications of biotransformation according to variants 2, 6 and 7 (Table 2). The results of enzymatic activity also confirmed the proper choice of the selected concentrations. Based on the results obtained after the first round of biotransformation in 8 different variants concentrations (Table 2), the medium composition was narrowed (Table 3) and subsequently the number of biotransformations carried out (Table 4). The reduced concentration of (NH_4_)H_2_PO_4_, KH_2_PO_4_, NaCl and the increased amount of glucose and other minerals components improved the biosynthesis of lactone **2a**. However, for bioreactor scale process further slight modifications in the concentration of medium components were done. The final cultivation medium applied in **B35** composed of (NH_4_)H_2_PO_4_ (7 g), KH_2_PO_4_ (3.5 g), glucose (50 g), NaCl (0.025 g), MgSO_4_ (1 g), CuSO_4_ (0.01 g), ZnSO_4_ (0.12 g), MnSO_4_ (0.02 g) per 1 L of H_2_O.

## Conclusion

In conclusion, the microbial stereoselective oxidation catalyzed by whole cell yeast was studied. Based on our previous results, *C*. *pelliculosa* ZP22 was selected to catalyze biotransformation of diol **3a** into (+)-(3a*S*,7a*R*)-isomer of lactone **2a**. A significant number of modifications concerning the temperature and composition of culture media were performed in order to improve lactone biosynthesis in the *C*. *pelliculosa* ZP22 culture in a bioreactor scale. Selection of the optimal medium was based on a two-level factorial design method. In all preparative experiments performed in a bioreactor, parameters like biomass, protein and enzymatic activity were under continuous control. The cultivation of *C*. *pelliculosa* ZP22 in a selected conditions (optimized medium composition and temperature 35 °C) applied in **B35** experiment afforded enantiomerically enriched (+)-(3a*S*,7a*R*)-isomer of **2a** (ee = 70%) in the 30% of isolated yield. Modification of medium composition and higher temperature of biotransformation increased the activity of pro-(+)-ADH in tested strain.

## Electronic supplementary material

Supplementary Information
