# Supplementary material for: Microbial synthesis of a useful optically active (+)-isomer of lactone with bicyclo[4.3.0]nonane structure
Source: Sci Rep. 2018 Jan 11;8:468. doi: 10.1038/s41598-017-18876-9 (PMC5765023; doi:10.1038/s41598-017-18876-9)
Supplement: Supplementary file 1 — Supplementary Information [file 41598_2017_18876_MOESM1_ESM.doc]

**Supplementary Information for publication**

**Microbial synthesis of a useful optically active (+)-isomer of lactone with bicyclo[4.3.0]nonane structure**

Filip Boratyński^1,*^, Agata Janik-Polanowicz^1^, Ewa Szczepańska^1^, and Teresa Olejniczak^1^

^1^Department of Chemistry, Wroclaw University of Environmental and Life Sciences, Wrocław, 50375, Poland

**^*^**filip.boratynski@upwr.edu.pl

[List of contents](#__RefHeading___Toc499645369)

[^1^H NMR spectrum of lactone **2a** (Supplementary Figure S1) 2](#__RefHeading___Toc499645370)

[^13^C NMR spectrum of lactone **2a** (Supplementary Figure S2) 3](#__RefHeading___Toc499645371)

[IR spectrum of lactone **2a** (Supplementary Figure S3) 4](#__RefHeading___Toc499645372)

[GC-MS spectrum of lactone **2a** (Supplementary Figure S4) 5](#__RefHeading___Toc499645373)

[Chiral Gas Chromatography (CGC) chromatograms of lactone **2a** (Supplementary Figure S5) 6](#__RefHeading___Toc499645374)


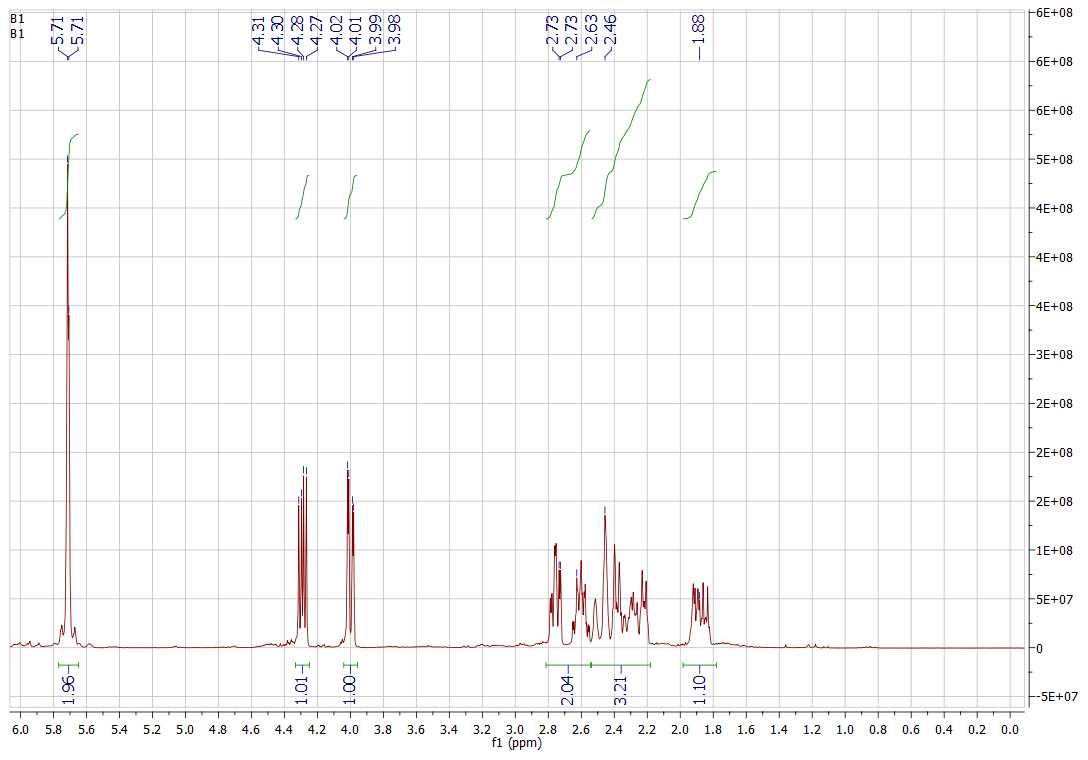


Supplementary Figure S1. ^1^H NMR (CDCl_3_, 600 MHz) spectrum of lactone **2a**.


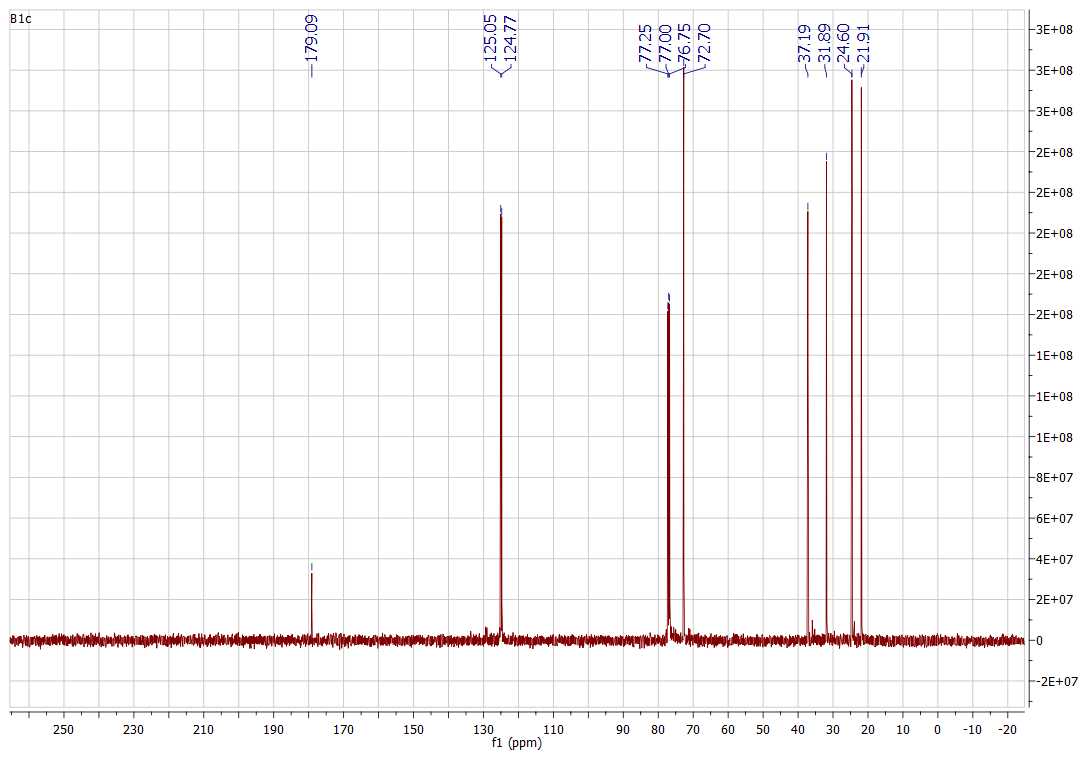


Supplementary Figure S2. ^13^C NMR (CDCl_3_, 151 MHz) spectrum of lactone **2a**.


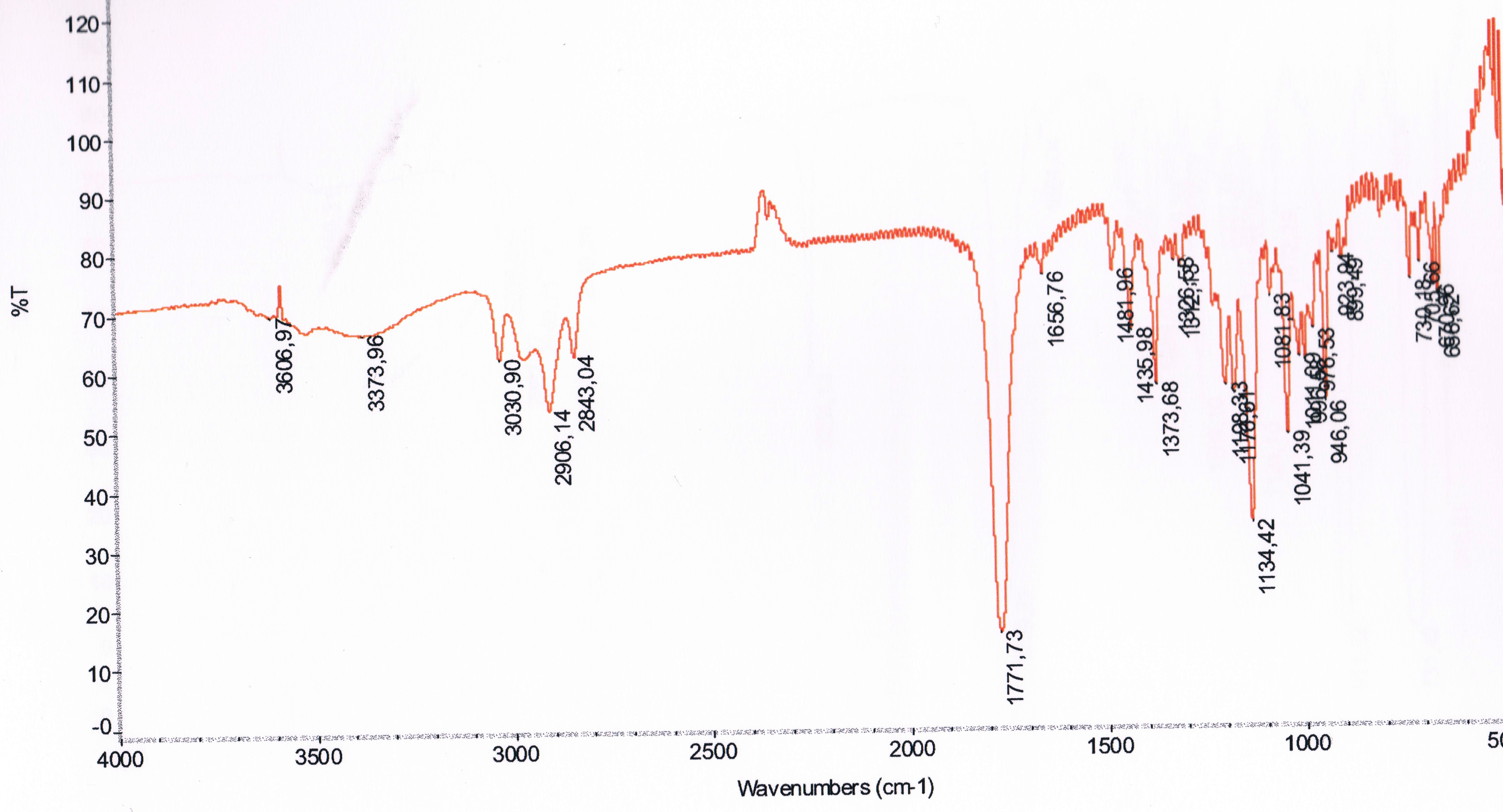


Supplementary Figure S3. IR spectrum of lactone **2a**.


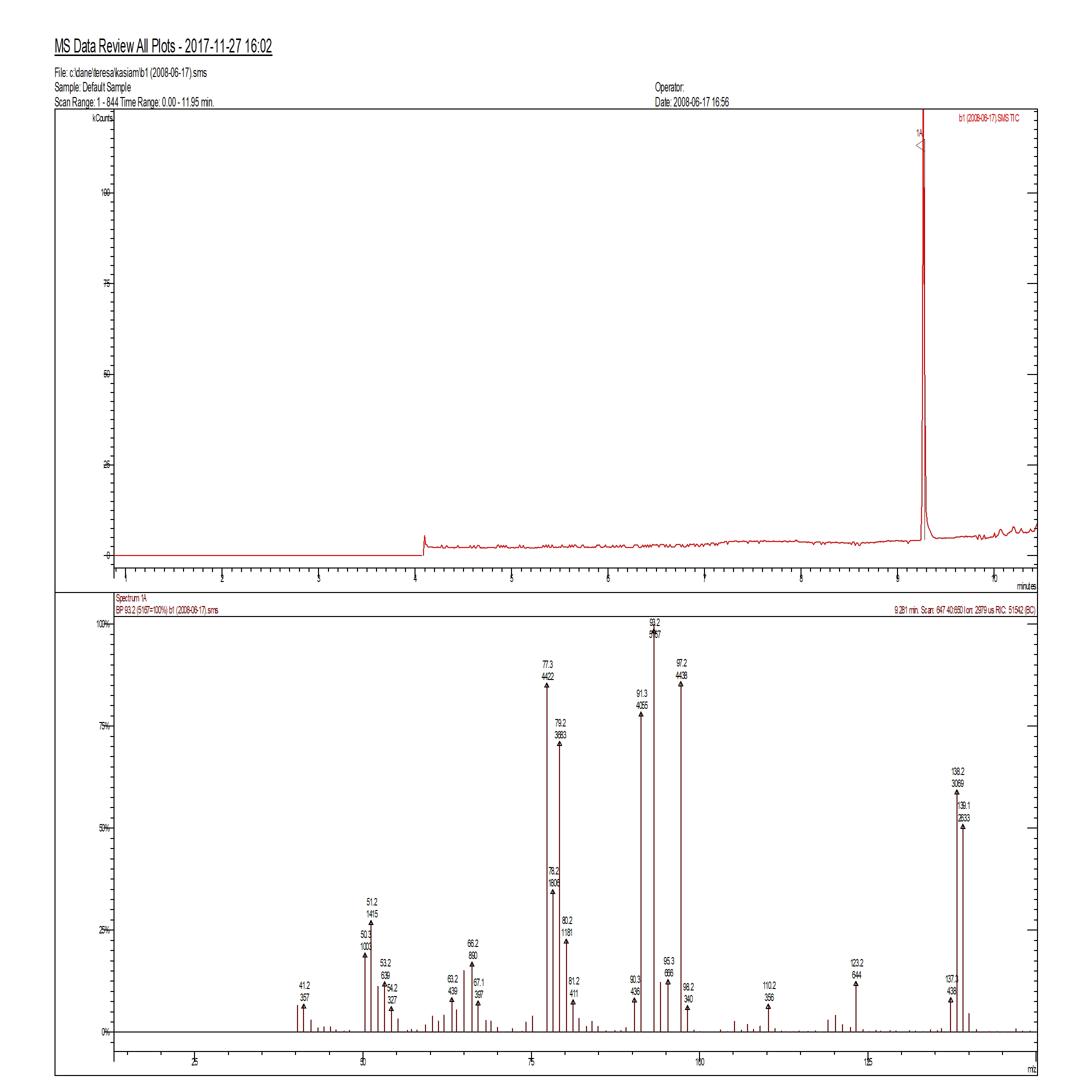


Supplementary Figure S4. GC-MS spectrum of lactone **2a**

Supplementary Figure S5. Chiral Gas Chromatography (CGC) chromatograms of lactone **2a**: (+)-(3a*S*,7a*R*)-isomer (ee = 70%) (top) and racemate (bottom).
